# Effectiveness and feasibility of self-sampling for human papillomavirus testing for internet-based cervical cancer screening

**DOI:** 10.3389/fpubh.2022.938272

**Published:** 2022-11-07

**Authors:** Jingran Li, Ruifang Wu, Xinfeng Qu, Xia Huang, Ling Li, Zhixin Lin, Zhijun Zhang, Jihong Deng, Rong Liu, Xiaofeng Zhao, Songling Zhang, Bei Lin, Ruifang An, Chao Zhao, Mingzhu Li, Yun Zhao, Lihui Wei

**Affiliations:** ^1^Department of Obstetrics and Gynecology, Peking University People's Hospital, Beijing, China; ^2^Department of Obstetrics and Gynecology, Peking University Shenzhen Hospital, Shenzhen, China; ^3^Department of Obstetrics and Gynecology, Jiangxi Maternal and Child Health Hospital, Nanchang, China; ^4^Department of Obstetrics and Gynecology, Maternal and Child Health Hospital of Guangxi Zhuang Autonomous Region, Nanning, China; ^5^Department of Obstetrics and Gynecology, Guizhou Provincial People's Hospital, Guiyang, China; ^6^Department of Obstetrics and Gynecology, Kunming City Maternal and Child Health Hospital, Kunming, China; ^7^Department of Obstetrics and Gynecology, Xining First People's Hospital, Xining, China; ^8^Department of Obstetrics and Gynecology, The Fourth Affiliated Hospital of Zhejiang University Medical College, Yiwu, China; ^9^Department of Obstetrics and Gynecology, The First Hospital of Jilin University, Changchun, China; ^10^Department of Obstetrics and Gynecology, Shengjing Hospital of China Medical University, Shenyang, China; ^11^Department of Obstetrics and Gynecology, First Affiliated Hospital of Xi'an Jiaotong University, Xi'an, China

**Keywords:** human papillomavirus, self-sampling, cervical cancer screening, internet-based, screening coverage

## Abstract

**Objectives:**

Worldwide, around 18.2% of cervical cancer occurred in China, mainly because of lower screening coverage and screening quality in regional disparities. To assess self-sampling for human papillomavirus (HPV) testing, combined with the internet, as a primary cervical cancer screening (CCS) method in low-resource settings, and to establish an internet-based self-sampling CCS-management model.

**Methods:**

The women who participated registered on a CCS website. We recruited 20,136 women, aged 30–59 years, from 13 provinces in China, to perform vaginal self-sampling for HPV testing as a primary CCS, based on the internet. A questionnaire was subsequently used to investigate the acceptability of self-sampling.

**Results:**

Of the 20,103 women with qualified samples, 35.80% lived in remote areas, 37.69% had never undergone CCS, 59.96% were under-screened, and the overall prevalence of a high-risk of HPV was 13.86%. Of 8,136 respondents, 95.97% of women felt that self-sampling was easy to perform, 84.61% had no discomfort when using a self-sampling brush, 62.37% women were more likely to choose self-sampling for CCS in future, and 92.53% were willing to introduce the concept to others around them. The reliability and ease of self-sampling were independent factors influencing selection of self-sampling (*p* < 0.05).

**Conclusions:**

The Internet-facilitated self-sampling for HPV testing and management model for cervical cancer prevention is feasible and effective. It can be used as a supplement to the conventional screening, particularly in outlying areas with few medical resources, to improve the coverage of CCS.

**Clinical trial registration:**

https://www.chictr.org.cn, identifier: ChiCTR2000032331.

## Introduction

Cervical cancer is the most diagnosed cancer and the leading cause of cancer death in women in developing countries (1, 2). As the second carcinoma on Chinese women following breast cancer, cervical cancer has been remaining the leading cause of cancer death of Chinese women under 50 of age for decades (3). It has been demonstrated that cervical cancer cannot be well-controlled without screening coverage of more than 70% (4, 5). In China, the cervical cancer screening (CCS) coverage from 2013 to 2014 ranged from 12 to 67% in different regions (6) even after the China national CCS program has been implemented for more than 12 years. Although some social element plays roles in unsatisfied cervical cancer prevention outcomes, it is clear that way of medical care provision to medically underserved women is one of the key issue for unsatisfied screening coverage and is the one that can be changed easily and feasibly at the time (4, 7, 8). It is obvious that inconvenience for participation is the main barrier for screening coverage because those programs were designed using provider sampling, which makes the screening programs can be conducted only in medical facilities and needs medically underserved women to go long distance for screening (6, 8, 9). High risk human papillomavirus (hr-HPV) had been demonstrated to be the necessary pathogen of cervical cancer, and many studies demonstrated that the high sensitivity of the HPV testing could maximize the effectiveness of CCS and its satisfied negative prediction value enables a 5–10 year of the screening interval (4, 5, 10, 11).

To make hr-HPV testing to be widely accessible in medically underserved remote area, investigators tried to study using self-collected samples for HPV primary testing 20 years. Since then, multiple studies have demonstrated that self-collected samples work as well as provider-collected samples when tested on polymerase chain reaction (PCR) based HPV testing (12–14). And it could remarkedly expand screening coverage in both urban and rural areas, since it is far less reliant on medical resources (11–13). Several preliminary research projects in China (8, 14–16) has verified that self-sampling offers possibility to make CCS reach under or non-screened women. Publications indicated that majority of cervical cancers occur in women who could not be regularly screened or properly treated for cervical pre-cancers due to less accessibility to qualified screening program (17–19). It is commonly recognized that women living in low-and-middle-income countries (LMIC), including China, should be the target population for expanding screening coverage. In year 2021, World Health Organization (WHO) initiated a global strategy by 2030 to scale up preventive, screening, and treatment interventions to eliminate cervical cancer as a global public heathy problem (18, 20). It is obvious that CCS is parallelly determinant with HPV vaccination to achieve WHO goals. Self-collected HPV testing has demonstrated to be the absolutely the effective way to make CCS to reach the medically underserved women living in remote area in the world. However, self-sampling does not just change the way of sampling but the manner of services. 8 years ago, Dr. Belinson and a group of Chinese investigators developed a community participatory self-sampling model for CCS and demonstrated that this model could potentially expanding screening coverage because it enabled primary screening be conducted without needs for involvement of medical providers in sampling procedures, in high efficiency in term of sampling, and at a very low rate of data error (21). Following the establishment of community participatory model (CPM) for CCS, Dr Wu, etc. (14) conducted a pilot study to have women apply primary screening on internet-based website. This was a small case size study but is highly valuable to contribute a solution for another barrier in cervical cancer screening activities, the data input. From then on, Chinese investigators have done a lot to develop internet-based platforms for cervical cancers screening and tried to know whether internet based screening is really work well-to improve the service efficiency, to simplify the screening procedures, and to increase the screening coverage.

We therefore designed and implemented this study to apply cervical cancer screening project in the remote rural communities in multiple provinces using self-collected HPV testing as the primary testing and adopting an internet platform for screening registration, purposing to investigate the key elements impacting the acceptability of self-sampling among women of different backgrounds and living in various communities, to identify the key determinants for setting up a self-sampling-based HPV-testing CCS project, to evaluate the effectiveness of self-sampling in terms of motivating project participation and expanding CCS coverage, and to verify the role of internet platform in the screening program.

## Methods

### Study populations

As a prospective study, the participants were recruited from remote towns, rural communities or town/city in 13 regions in China, including Beijing, Liaoning Province, Jilin Province, Shaanxi Province, Qinghai Province, Zhejiang Province, Guangdong Province, Jiangxi Province, Guizhou Province, Yunnan Province, the Inner Mongolia Autonomous Region and the Guangxi Autonomous Region in China. The target communities distributed in Northern, Southern, Middle, Southwest, and West of China. The inclusion criteria were: (1) 30 to 59 years of age, (2) sexually exposed, (3) non-pregnant, and (4) consent for participation. The exclusion criteria were: (1) had cervical surgical history as cold knife conization (CKC) and loop electrosurgical excision procedure (LEEP), (2) was performed hysterectomy or pelvic radiotherapy, and /or (3) was suffering acute or recurrent genital and urinary tract infections.

**Figure 1:**
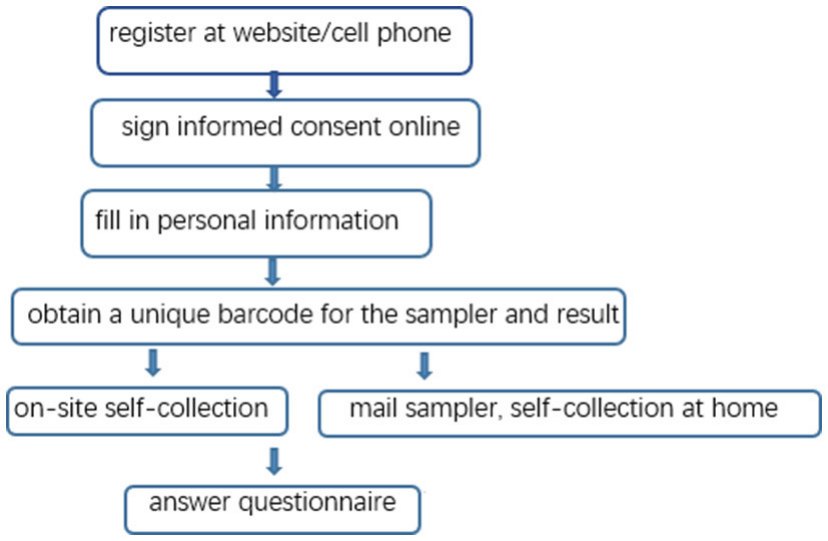
Working flowchart for the study.

Recruitment notifications were released via several available public or inter-personal ways as the website, WeChat moment, and telephone call, followed by sending an oral explanation or an introductive printings by the community workers. Women were recruited for participation by medical staff from the community medical centers, the local maternal and child health systems, the local hospitals, the local branch of Women's Federation, and sub-district offices or community service. Women who were willing to participate needed to visit any of the screening sites nearby for registration. They were encouraged to registered for participation through signing up the website (http://47.106.227.241/) via their personal computers or smartphones. Any woman who needs help in registration online can search for assistance from staff at the sites to accomplish the registration. Successful registration required eligible women to fill out a personal information form and to sign an electronic version of informed consent form. Also, a written informed consent form was provided for the offline signature of uneducated women by fingerprints with the witness of a third person who had not involved in the screening project. Successfully registered participation was subsequently provided with a sampling kit for self-sampling. The working flowchart for the study was shown in Figure 1.

This study was organized by Peking University People's Hospital (PKUPH) and Peking University Shenzhen Hospital (PSUH) and was conducted from September 2018 to July 2020. Before implementation, it had been approved by the Ethics Committee of Peking University People's Hospital (2018PHB056-01) and registered on the Chinese Clinical Trial Website (https://www.chictr.org.cn, ChiCTR2000032331).

### Self-collection of the vaginal samples

The sampling kit provided to the women contained a cone-size brush, a sample processing card (FTA based card, BGI-Shenzhen, China) or a vial containing preservative solution (Bioperfectus, Taizhou, China), a graphic/textual sampling instruction and several copies of a unique barcode that was not only the sample identification code but also the patients study ID for further diagnostic examination when tested positive. All participants were guided to collect their own vaginal samples in referring to the sampling instruction or the interpretation of the local staff on sites, No assistance would be provided to any women when they perform self-sampling.

When sampling, participant would take a squat or a standing position with one feet on a bench to open the legs, hold the handle of the sampling brush to insert the head of the brush into vagina, shaking back and forth aligning the axes while pushing the brush inward, until feeling resistance. This was followed by rotating the brush up to five times prior to removing it from the vagina. After sampling, women who got a sample processing card would apply the sample on the brush-head on the sample application area of the card until they saw color changes, while women who got a vial would place the brush-head into the vial and cover it by screwing the cap of the vial tightly. An on-site nurse or women themselves would check each vial to confirm that the brush-head was in the vial and that none of the solution had leaked out.

### HPV testing

The self-collected samples on card were tested on SeqHPV(BGI-Shenzhen, China) and that in liquid vials were tested on BMRT (BioPerfectus, China). We used FTA card to process samples for SeqHPV and liquid vial to process samples for BMRT test. SeqHPV is a sequencing-based HPV DNA assay developed by BGI Genomics (Shenzhen, China). It amplifies HPV DNA by multiplex real-time PCR and determines HPV genotypes (if any) by new generation of sequencing (NGS). With sequencing the E6/E7 DNA at L1, it reports 14 h-HPV genotypes (HPV16, 18, 31, 33, 35, 39, 45, 51, 52, 56, 58, 59, 66, and 68). It has been validated in multiple trials to work well with both self- and provider-collected samples processed in liquid media and on an FTA^®^ card (22). It adopted quality control in three testing procedures: (1) DNA extraction, in which a negative standard substance was added to every 30 samples to monitor any contamination; (2) PCR, in which five negative substances (purified water) and a positive reference (plasmid quality control) were added to every 96 samples to monitor the PCR process; and (3) HPV genotyping, in which the target peak from Agilent 2100 Bioanalyzer and the concentration of Q-PCR quantification be would be used to confirm the quality of the library. BMRT, The BioPerfectus Multiplex Real-Time PCR assay, is a fluorescence-based quantitative HPV testing assay developed by Bioperfectus Technologies Co., Ltd. (Jiangsu, China). BMRT reports 21 HPV genotypes, including 14 h-HPV as same as SeqHPV and HPV 6, 11, 26, 73, 81, and 82 (23). It has been validated to work well-with both self- and provider- collected samples in liquid media as it is also a PCR-bases assay. As there is no strong evidence to show the pathogenic relation between low-risk HPV and cervical cancer, to make the testing results from the two assay comparable, we will just analyze BMRT results related to hr-HPV and treat the cases positive of low-risk HPV as negative of hr-HPV.

### Colposcopy and biopsies

Women who were primarily tested positive for any of the 14 h-HPV types were referred for colposcopy. Multiple biopsies were taken for all patients from any colposcopically suspected lesion site or, if no suspected lesion site was confirmed, at the transformation zone on four quadrants of the cervix. Endocervical curettage (ECC) was performed on any patients whose TZ could not be completely visible under colposcopy. Histological analysis was conducted by pathologists from PKUPH and diagnostic results were reported as high-grade squamous intraepithelial lesions (HSIL), low-grade squamous intraepithelial lesions (LSIL), and cervical mucositis, which served as the gold referance for evaluation the screening effects.

### Statistical analysis

Statistical analyses were performed using SPSS software (version 20.0) for Windows (IBM SPSS, Inc., Armonk, NY, USA) and Excel (version 2013; Microsoft, Redmond, WA, USA). For analysis of the data regarding to socio-demographics and women's attitudes to self-sampling, participants were grouped according to age, educational background, marital status, income, service accessibility, and screening history. The count data were subjected to chi-square testing. *P*-values were two-sided, and *P* < 0.05 was considered statistically significant.

## Results

A total of 20,136 participants were enrolled for the primary screening of the study and provided self-collected vaginal samples. After excluding 10 (0.05%) for labeling errors and 23 (0.11%) for unqualified samples, 20,103(99.89%) participants had results for HPV testing on either SeqHPV or BMRT (the screened women). Among those, 2,787 (13.9%) women were tested positive of hr-HPV, of whom, 2,045 women (73.38%) returned for colpo-biopsies with pathological outcomes. As this analysis will just focus on the screening process, we included all screened women into the data set.

### Socio-demographic characteristics

The mean age of the participants who had primary testing (the screened women) was 44.31 ± 7.70 years. Of those screened women, 35.80% (7,198/20,103) were from remote areas [defined as rural communities that were more than 10 kilometers away from hospitals providing cervical cancer prevention services (PCPS-hospitals)], while 43.54% were living in towns (defined as in town/city communities that were < 5 kilometers away from PCPS-hospitals), and 19.18% were from suburban communities (communities that were 5–10 kilometers away from PCPS-hospitals). Socio-demographic data are listed in Table 1.

**Table 1:** Demographic and behavioral features.

**Item**	**Cases**	**%**	**Item**	**Cases**	**%**
**Age (year)**	19,873				
30–34	2,610	13.13	< 5,000	9,352	58.21
35–39	3,448	17.35	**Gravidity and parity**	16,106	
40–44	3,814	19.19	0	351	2.18
45–49	4,322	21.45	1–2	11,603	72.04
50–54	3,485	17.54	3 times and above	4,152	25.78
55–59	2,194	11.04	**Screening history**	16,155	
**Education**	16,137		Never screened	6,089	37.69
College and above	6,980	43.25	Under screened	9,686	59.96
Middle school	6,165	38.20	Regularly screened	380	2.35
Primary school and below	2,992	18.54	**Past screening methods**	9,827	
**Occupation**	16,126		Base-HPV	3,222	32.79
Civil servant/public institution personnel	4,202	26.06	Base-Cyto	6,605	67.21
Company white-collar	1,617	10.03	**Start of sex**	16,095	
Migrant labor	2,877	17.84	≤ 20	2,396	14.89
Peasant	2,035	12.62	21–25	10,003	62.15
Inoccupation	1,711	10.61	above 25	3,696	22.96
Other	3,682	22.83	**Number of sexual partners**	16,084	
**Monthly family income (RMB)**	16,066		1	15,391	95.69
> 30,000	346	2.15	2–3	357	2.22
10,000–30,000	1,344	8.37	4 and above	32	0.20
5,000– < 10,000	5,024	31.27	Unwilling to answer	304	1.89

The overall rate of hr-HPV infection was 13.86%. The top five most prevalent HR-HPV subtypes were HPV52 (3.42%), HPV58 (2.29%), HPV16 (2.17%), HPV39 (1.35%), and HPV51 (1.30%).

### Acceptability of self-sampling

Of the screened women, 8,136 responded to questionnaires (the respondents), but not all of them completed all questions. Of the respondents, 95.97% (7,080/8,136) responded “feeling self-collection is easy to do,” and 84.61% (6,884/8,136 responded “no discomfort when using the self-sampling brush.” Moreover, 62.37% (5,074/8,136) of the respondents expressed their preference for using self-sampling for CCS in future. Those numbers are quite encouraging to the investigators for making self-sampling widely adopted in cervical cancer screening programs projected to cover more women living in remote regions. The percentage and number of women who specified reasons for self-sampling preference are listed in Table 2.

**Table 2:** The participants' recognized reasons for attitude to provide sampling.

**Attitude of participants for provider sampling**	***N***	**%**
More accurate for testing	2,032	42.03
More reliable results	1,621	33.53
Other problems detectable while sampling	900	18.61
Traditionally sample should be collected by provider	213	4.41
Others	69	1.43
Total	4,835	100.00
**Attitude of participants for self-sampling**		
More private	1,771	21.84
More convenient	2,647	32.65
Easier to operate	1,405	7.33
Less costly	427	5.82
• Less painful • Others	• 1,717 • 142	• 21.18 • 1.74
Total	8,108	100.00

**Figure 2:**
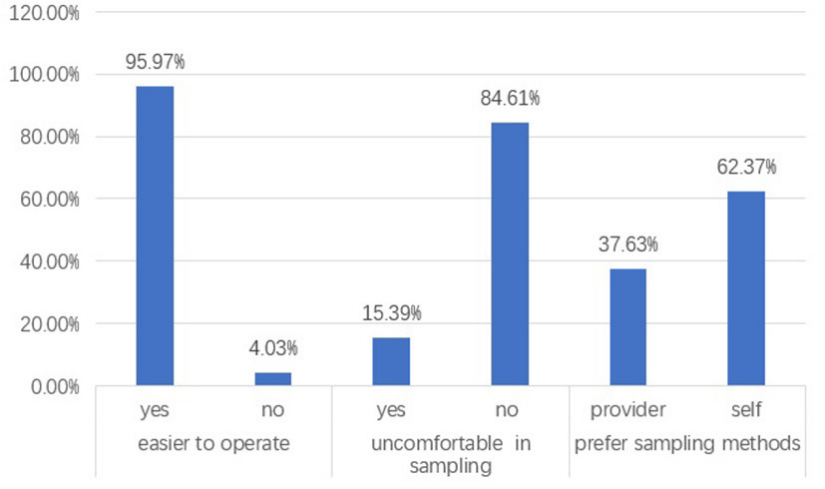
Results from survey on the participant's acceptance of and preference for self-sampling.

Of the respondents, 5,842 (71.80%) responded to the questions regarding their preference to self-sampling sits, and 51.65% (3,017/5.842) of them choose “self-sampling at hospital,” 38.30% (2,237/5,842) choose “self-sampling at home,” and 5.19% (303/5,842) choose “self-sampling at nearby community healthcare centers or clinics,” indicating that inconvenience in access to screening services might be a significant barrier of community women to their participation in hospital-centralized screening projects. Another fact to show the acceptance of community women to self-sampling is that 92.53% (5,406/5,842) of the respondents expressed their willingness to introduce self-sampling for HPV testing to others. Participants' acceptance of and perception after self-sampling are shown in Figure 2 and Table 3.

**Table 3:** Participant's stated reasons for choosing self-sampling.

		***N* (%)**
**Site for self-sampling**		
	At home	2,237 (38.30)
	In hospital	3,017 (51.65)
	Community site/nearby clinic	303 (5.19)
**Reliability of the results from self-sampling**		
	0–20	136 (1.87)
	21–40	656 (9.03)
	41–60	1,094 (15.05)
	61–80	3,228 (44.42)
	81–100	2,153 (29.63)
**Amount willing to pay for self-sampling base screening (RMB)**		
	< 50	4,367 (54.55)
	50–100	1,931 (24.12)
	101–150	997 (12.45)
	151–200	536 (6.70)
	>200	174 (2.17)
**Wished-for financial resources for self-sampling-based screening**		
	Government	5,282 (63.40)
	Social Insurance	2,145 (25.75)
	Medical services	666 (8.00)
	At one's own expense	156 (1.87)
	Others	82 (0.98)
**Willingness to introduce self-sampling to friends and relatives**		
	Willing	7,430 (92.53)
	Unwilling	262 (3.26)
	Not sure	338 (4.21)
**What is the greatest concern regarding application for screening via internet**		
**and self-sampling at home**		
	Reliability of internet services	2,131 (20.54)
	Reliability of testing result	3,086 (29.75)
	Potential deterioration of contamination on samples during transportation	2,983 (28.76)
	In-time result reporting	1,917 (18.48)
	Others	256 (2.47)

Multivariate analysis showed that the reliability of self-sampling and its easiness were independent factors influencing self-sampling preferences (*p* < 0.05). No statistical significance was found in different groups of age, education, occupation, gravidity, medical insurance, or age of sexual initiation (*p* > 0.05) (Table 4). Analysis on the factors influencing participants' preference for self-sampling or provider-sampling shows that self-sampling preference was related to education level, occupation, age of sex-exposure, availability of social insurance, and realization to the easiness and reliability of self-sampling.

**Table 4:** Multivariate analysis of the factors influencing self-sampling tendency (Binary logistic regression analysis).

		**B**	**S.E**.	**Wald**	***p***	**Exp(B)** **(OR)**	**95%CI**	
Intercept		−3.672	1.434	6.561	0.010			
Reliability of self-sampling (%)	0–20	0.275	0.175	2.466	0.116	1.317	0.934	1.856
	21–40	0.578	0.195	8.831	0.003	1.783	1.218	2.611
	41–60	0.350	0.159	4.844	0.028	1.419	1.039	1.937
	61–80	−0.407	0.124	10.825	0.001	0.666	0.522	0.848
	81–100	0^a^	.	.	.	.	.	.
Age groups (years)	30–34	0.231	0.197	1.372	0.241	1.260	0.856	1.855
	35–39	−0.251	0.179	1.968	0.161	0.778	0.548	1.105
	40–44	−0.037	0.179	0.042	0.838	0.964	0.678	1.370
	45–49	−0.251	0.173	2.095	0.148	0.778	0.554	1.093
	50–54	−0.238	0.175	1.842	0.175	0.788	0.559	1.111
	55–59	0^a^	.	.	.	.	.	.
Social insurance	Yes	0.049	0.633	0.006	0.938	1.051	0.304	3.629
	No	−0.046	0.604	0.006	0.939	0.955	0.292	3.120
	In-patient	0^a^	.	.	.	.	.	.
Ease/difficulty of self-sampling	Easy	4.946	1.014	23.818	0.000	140.657	19.296	1025.327
	Hard	0^a^	.	.	.	.	.	.
Age at first sex exposure (years)	≤ 20	0.260	0.175	2.204	0.138	1.297	0.920	1.828
	21–25	0.031	0.124	0.064	0.801	1.032	0.809	1.316
	≥26	0^a^	.	.	.	.	.	.
Education	Primary and lower	0.059	0.274	0.046	0.831	1.060	0.620	1.813
	Middle	0.077	0.246	0.097	0.755	1.080	0.667	1.748
	University	0–0.063	0.218	0.082	0.774	0.939	0.612	1.441
	Master and above	0^a^	.	.	.	.	.	.
Occupation	Unemployed	0.204	0.270	0.572	0.450	1.227	0.722	2.083
	Peasant	0.337	0.204	2.734	0.098	1.401	0.939	2.089
	Worker	0.066	0.170	0.151	0.698	1.068	0.766	1.489
	Office Lady-industrial	0.174	0.197	0.774	0.379	1.190	0.808	1.752
	Civil Servant or clerk	0.075	0.154	0.237	0.626	1.078	0.797	1.458
	Student	0.072	0.295	0.059	0.807	1.075	0.603	1.914
	Other	0^a^	.	.	.	.	.	.

a, In each indicator, the data in the last row is used as the standard, and the other indicators are compared with the indicators in the last row.

OR, odds ratio; CI, confidence interval.

The number of women who felt self-sampling be easy to do was significantly higher than that of women who thought self-sampling be difficult and who felt provider-collection be easier (*p* < 0.001). Self-sampling was significantly preferred by most women at all education background (*p* < 0.001) and all kinds of occupations (*p* < 0.001), without effect from social medical insurance availability and irrespective of parity from sex exposure age and the scale given to self-sampling (*p* < 0.001). No difference in sampling preference was observed regarding marital status, residence category, contraception, or screening histories (Table 5).

**Table 5:** Analysis of elements influencing the choices for self- or provider-based sampling.

		**Self-sampling preferable (*n* = 3,551)**	**Provider-collection preferable** **(*n* = 1,891)**	**X^2^**	***P***
**Ease of sample collection**					
	Easy	3,309 (93.4)	1,878 (99.7)	117.337	< 0.001
	Difficult	234 (6.6)	5 (0.3)		
**Education**					
	Primary school and below	557 (18.6)	290 (19.0)	23.189	< 0.001
	Middle school	1,271 (42.5)	628 (41.1)		
	College and above	1,160 (38.8)	609 (39.9)		
**Marital status**					
	Married	2,926 (97.6)	1,501 (97.9)	4.900	0.180
	Unmarried	15 (0.5)	13 (0.8)		
	Divorced	47 (1.6)	15 (1.0)		
	Widowed	11 (0.4)	4 (0.3)		
**Occupation**					
	Unemployed	343 (11.4)	141 (9.2)	109.665	< 0.001
	Farmer	424 (14.2)	151 (9.9)		
	Migrant worker	373 (12.5)	362 (23.8)		
	Office worker	292 (9.8)	124 (8.1)		
	Civil servant/public institution personnel	784 (26.2)	413 (27.1)		
	Other	771 (25.8)	333 (21.9)		
**Census registered**					
	Yes	2,664 (88.6)	1,352 (87.6)	1.08	0.582
	Residence	321 (10.7)	178 (11.5)		
	Temporary residence	21 (0.7)	13 (0.8)		
**Medical insurance**					
	Yes	2,834 (94.5)	1,392 (90.6)	31.054	< 0.001
	No	135 (4.5)	132 (8.6)		
	For inpatient only	30 (1.0)	12 (0.8)		
**Number of births**					
	1–2	2,129 (71.3)	1,153 (75.7)	10.389	0.006
	≥3	813 (27.2)	356 (23.4)		
	0	42 (1.4)	14 (0.9)		
**Contraception**					
	Tools	750 (25.2)	375 (24.7)	4.198	0.280
	IUD	630 (21.2)	314 (20.7)		
	Oral contraceptive	152 (5.1)	91 (6.0)		
	No	770 (75.9)	421 (27.7)		
	Other	674 (22.6)	319 (21.0)		
**History of screening**					
	Never	911 (30.5)	487 (31.7)	5.328	0.07
	Under-screening	1,995 (66.7)	1,024 (66.6)		
	Regular screening	85 (2.8)	27 (1.8)		
**Age of becoming sexually active (years)**					
	≤ 20	411 (13.7)	273 (17.8)	13.642	0.001
	21–25	1,973 (65.9)	950 (62.1)		
	≥26	610 (20.4)	308 (20.1)		
**Reliability scale for self-sampling (%)**					
	0–20	384 (10.9)	225 (12.3)	71.556	< 0.001
	21–40	236 (6.7)	190 (10.4)		
	41–60	408 (11.6)	300 (16.4)		
	61–80	1,519 (43.2)	613 (33.4)		
	81–100	966 (27.5)	505 (27.6)		

## Discussion

It has been reported that there are many factors impacting the lower coverage of CCS (6, 9, 24, 25). The elements that impact screening coverage and can be feasibly improved are the technologies used in primary screening tests, the way of delivering screening services, and the roles of medical providers. Self-sampling based on community screening with internet facilitation can provide convenient access for women living in medically underserviced regions and may potentially provide a solution for extending CCS to cover the majority of women in the world.

With the giant population, Chine contributes a big part of the cervical cancer cases and deaths. As one of the LMICs, China still has big portion of women living in medically underserved region in which screening programs strongly relying medical resourced in primary screening cannot reach most of women in those area. According to China Cancer Reports, cervical cancer is most prevalent in women at age of 45–64, and it is reported by variety of studies that most cervical precancers (CIN2/3) are detected in women at age of 30–59, indicating that the most sensitive age span for pre-cancer prevalence is 30–59 (3, 7, 9).

Using the validated self-sampling technology and the HPV testing assays that has been validated to work well-with self-sampling, we recruited women at the most sensitive age for precancers and living in variety of communities to verify the effectiveness and feasibility of self-sampling in community cervical cancer screening demonstrated that self-sampling is widely accepted by community women regardless education, occupation, sexual experience, and ages.

### The questionnaires to investigate participant's attitude to internet facilitated self-HPV screening

The questionnaires we provided to the participants included 10 questions, followed by 50 selectable choices related to three aspects: (1) experience of self-sampling; (2) preference for sampling methods, and (3) self-perception on self-sampling. The questions were designed to survey the public awareness of CCS and medical services and the public concept to cancer prevention. Based on the results of the questionnaire survey, most of the respondents, regardless education, occupation, and social insurance, feel self-sampling be easy to do without obvious discomfort and expressed their interests in self-sampling for their future CCS. As none of the questions showed feelable comparison of self- or provider-sampling, this result can represent the direct perception of those women to the two kinds of sampling and indicates that there is no significant objective barrier to replace provider-sampling with self-sampling in HPV testing based primary screening. However, the responses reflect the strong trust of the public on medical facilities and self-sampling, which might be suggestive evidence to introduce self-sampling into medical facilities.

### Feasibility and acceptability of self-sampling

Internet services have covered most areas of China, including the vast rural regions, which is the basis for adoption of internet to facilitate CCS. Public education for CCS through the internet can reach most women with access to internet services. In our study, 35.80% of the responders were living in outlying communities, 89.48% were from low-income families, 18.54% were backgrounded with primary, lower education, or illiteracy. As most of those women would have no barrier to accept self-collected HPV testing based screening and most of the areas they are living in are covered with internet services which have been accepted to be the dominant life style of those women, internet-facilitated self-sampling-based screening would be completely feasible and applicable in outlying regions and to poorly educated and low-income women (2, 14, 19), which is able to provide opportunities to women who do not have access to provider-sampling-based CCS and, therefore, to expand the coverage of the screening. In our study, 37.69% of participants reported never being screened. This percentage was significantly higher than those who reported being screened regularly. This indicates that our project, which was based on self-sampling and facilitated with internet services, created access to screening for many women who were never screened, and suggests that screening program should be designed feasible to target women who have never been screened.

Many studies have demonstrated that self-sampling had high acceptability among women of various spheres, given that it is attractive for its convenience of performing, enabling sampling at home, and less discomfort, embarrassment, pain, and anxiety (26–29). In our study, the post-sampling survey showed good acceptance of self-sampling after the women had experienced it. In addition, 78.67% of the respondents expressed their acceptance of paying < $16 (equaling RMB 100) for self-collected screening, indicating that self-sampling-based screening programs may potentially be able to reach the WHO recommended coverage (70%) if the price can be controlled under $15 per case.

Since self-sampling is easy to learn and can be mastered by women in general (21, 26, 27), it is possible to organize a screening project via internet services. With internet service, women can participate in CCS at home or at facilities nearby, without the need to travel a distance to find medical services and to spend time simply to wait for sampling, which, together with the lack of need for a doctor's involvement in the sampling, is obviously cost-effective. Self-sampling-based HPV testing can increase participation and therefore increase the coverage of CCS (11–14). In addition, privacy protection and the convenience of self-sampling will also encourage office workers to participate in the screening program. In our study, 43.25% of the participants had educational background of university level and above, 36.09% were office workers and public servants. Multi-variant analysis showed that there was no significant influence of different occupations, education levels, ages, medical insurance types, or the age of becoming sexually active on the acceptance of self-sampling. This indicates that self-sampling is suitable for most women, regardless of their background and demographic differences. Moreover, 64.11% of respondents expressed willingness to introduce self-sampling to their relatives and friends, suggested the possibility of popularization of self-sampling.

Self-sampling does have barriers in terms of application. Based on our study, those barriers are mostly cognitive. Multi-variant analysis showed that the top independent reason for not choosing self-sampling was “not trusting the test result” (29.75%), followed by “worrying about specimen contamination during shipment” (28.76%). Both the reasons were cognitive, related to information asymmetry, but not based on evidence. Another independent reason was that self-sampling was “hard to do,” as indicated by some respondents. However, we currently cannot confirm that this is an experience-based answer, because we have no evidence regarding how many respondents who gave that answer had never been screened via provider-based sampling. Other reasons given for not choosing self-sampling were all cognitive. Overall, self-sampling was associated with high confidence and acceptability (28).

Self-sampling can allow CCS be performed in medically underserviced regions and populations at an affordable cost (30, 31). However, self-sampling-based screening programs requires the participants, the community, and the medical providers to play different roles in primary screening, positive triage, and pre-cancer treatment. Internet services could be the most effective platform to link these components to play their respective roles (14). Internet services can also play important roles in public education and participation motivation.

In addition, there are some limits in this study. Firstly, the designed questionnaire only includes questions raised by researchers, which is not well-targeted, and individual interviews are not conducted. Secondly, If there are problems among women sampled at home, consultation is not convenient. Another, if are there any different and influencing factor of the acceptability in the group with higher health literacy or consciousness and with lower levels of health literacy need to further analysis.

In conclusion, the internet-facilitated self-sampling-based HPV-testing for CCS and management model for cervical cancer prevention, in a large sample, is feasible and effective. This approach can be used as a supplement to traditional screening in China, particularly in outlying areas with few medical resources, which will result in significant improvement of the coverage of CCS.

## Data availability statement

The raw data supporting the conclusions of this article will be made available by the authors, without undue reservation.

## Ethics statement

The studies involving human participants were reviewed and approved by Peking University People's Hospital. The patients/participants provided their electronic version of informed consent to participate in this study.

## Author contributions

JL, RW, and LW contributed to the study's conception, design, analysis, and interpreted the data. RW, XQ, and LW provided important suggestions in analysis and writing of the paper. All authors carried out data collection in the study, read, and approved the final version of the paper.

## Funding

This work was supported by the Association for Maternal and Child Health Studies (2018AMCHS00801) and the National Key Research and Development Program of China (2016 YFC1302901).

## Conflict of interest

The authors declare that the research was conducted in the absence of any commercial or financial relationships that could be construed as a potential conflict of interest.

## Publisher's note

All claims expressed in this article are solely those of the authors and do not necessarily represent those of their affiliated organizations, or those of the publisher, the editors and the reviewers. Any product that may be evaluated in this article, or claim that may be made by its manufacturer, is not guaranteed or endorsed by the publisher.
